# Management of pleural empyema in a 12-year-old obese patient with COVID-19: a pediatric case report

**DOI:** 10.1186/s12887-021-03007-1

**Published:** 2021-11-30

**Authors:** Reza Abbasi, Farnaz Sadat Javanmardi, Ahmad Mokhtari, Parisa Hosseinpour, Reza Shahriarirad, Kamyar Ebrahimi

**Affiliations:** 1 Department of Pediatrics, Faculty of Medicine, Yasuj University of Medical Sciences, Yasuj, Iran; 2 Department of Internal Medicine, Faculty of Medicine, Yasuj University of Medical Sciences, Yasuj, Iran; 3 School of Medicine, Islamic Azad University, Kazeroun branch, Kazeroun, Iran; 4 Student Research Committee, Shiraz University of Medical Sciences, Shiraz, Iran; 5 Thoracic and Vascular Surgery Research Center, Shiraz University of Medical Sciences, Shiraz, Iran

**Keywords:** COVID-19, Pediatrics, Dyspnea, Empyema, Coronavirus, Obese

## Abstract

**Background:**

With the ongoing coronavirus disease (COVID-19) pandemic, along with the development of new mutations of the virus and an increase in the number of cases among pediatrics, physicians should be aware and alerted on the atypical presentations of the disease, especially in less expected individuals.

**Case presentation:**

Here we present a 12-year-old obese boy (BMI = 37.5 kg/m^2^) who presented with empyema, which was following SARS-CoV-2 infection. The patient had no history of fever. Due to the onset of dyspnea, a chest tube was inserted for him which was later altered to a pleural drainage needle catheter.

**Conclusion:**

Our case is the first report of COVID-19 presenting as empyema among pediatrics. Pleural empyema should be considered as a rare complication of COVID-19. Since there is still no guideline in the management of empyema in the context of COVID-19, delay in diagnosis and intervention may cause morbidity and mortality in children.

## Background

The Coronavirus disease of 2019 (COVID-19) pandemic, caused by the severe acute respiratory syndrome coronavirus-2 (SARS-CoV-2), has become a global concern and epidemiological threat [1]. Patients may have a wide range of manifestations from asymptomatic or having symptoms such as dry cough, fever, muscle weakness, chest pain, and respiratory distress [1]. However, the severity of the symptoms varies from person to person and even some may manifest as atypical symptoms [2]. Research has shown that COVID-19 not only affects adults, but children too. The disease is usually asymptomatic in pediatrics and rarely progresses to hospitalization or mortality, while COVID-19 is much more frequent in adults than children, similar to many previous studies [1, 3–5]. Furthermore, In contrast with adults, comorbidities and underlying diseases are not common in children [6]. The disease impacted many countries and populations and necessitated alterations in lifestyles and policies [7–12]. while there are still issues regarding the prompt management, diagnosis, and treatment of the disease [13–18]. Moreover, new mutations in the COVID-19 genome appear to have more mortality and morbidity in younger aged individuals [19]. Furthermore, approximately half of children’s cases are asymptomatic or mild cases, which accounts for misdiagnosis of the disease [20].

Despite the process of worldwide vaccination, in some countries (such as Iran), individuals under the age of 18 are not yet concerned as a priority in vaccination, due to insufficient data regarding its efficacy and safety, although they can be infected and encounter morbidity and mortality [21, 22]. Furthermore, in other countries such as the United States, Europe, and Israel, in which COVID-19 vaccination of the youth has been performed, there still is a long way until the entire juvenile population is vaccinated and protected. Even so, with alterations and mutations concurring in the virus, rapid reporting of the different manifestations is essential to provide prompt diagnosis and management, and decrease the complications and spread of the disease. Thus, more attention is needed in younger individuals contracting COVID-19 to prevent severe cases of infections. Here we present a 12-year-old boy who presented with an atypical manifestation of COVID-19. Based on literature, our case is the first report of COVID-19 presenting as empyema among pediatrics.

## Case presentation

The patient is a 12-year-old male with flu-like symptoms including dry cough and rhinorrhea for three weeks before his admission at our center. His parents stated that the patient has had no fever or chills since his symptoms started. They also mentioned that the patient and his parents all had flu-like symptoms for the past 3 weeks, which resolved for his parents, but the patient’s symptoms became more severe. Based on the patient and his parents’ past medical history, he had no history of recurrent respiratory infections, hospitalization, immunodeficiency, recurrent pneumonia or otitis media, sepsis, or contact with a patient suspected or infected with tuberculosis.

The patient was then visited by his doctor who prescribed Azithromycin (500 mg per day) for 5 days, and then due to no improvement was changed to co-Amoxiclav (Amoxicillin-Clavulanic acid, 625 mg every 8 h) for 7 days. However, no improvement was achieved.

After three weeks, the patient was brought to our center due to the progression of his symptoms and developing dyspnea. In our initial evaluation, the patient’s symptoms were severe coughs, nasal flaring, respiratory distress with a respiratory rate of 35, and decreased O_2_ saturation (85%). He was also afebrile. Significant findings in his initial physical exam included obesity (Weight = 96Kg, Height = 160 cm, Body Mass Index = 37.5 kg/m^2^) and no breathing sound in his left lung.

His initial laboratory test results showed an increased White Blood Cells (W.B.C) count (19.2 × 10^3^/mm^3^) with a lymphocyte differentiation of 16%, high platelet (Plt) count (593 × 10^3^/mm^3^), high Erythrocyte Sedimentation Rate (ESR) (80 mm/h), and a slightly increased amount (+ 1) of C-Reactive Protein (CRP; evaluated with Bionic CRP kit, Tehran, Iran) which suggests that the patient had an active inflammation. The patient also had a positive D-Dimer test (0.6 mcg/ml) and a low hemoglobin level (11.6 g/dl). The results of his Ventricular Blood Gas (VBG) were also suggestive of a primary respiratory alkalosis (pH = 7.46, pCO2 = 29.5, HCO3 = 21.3 mmol/L). Renal tests were unremarkable.

**Fig. 1:**
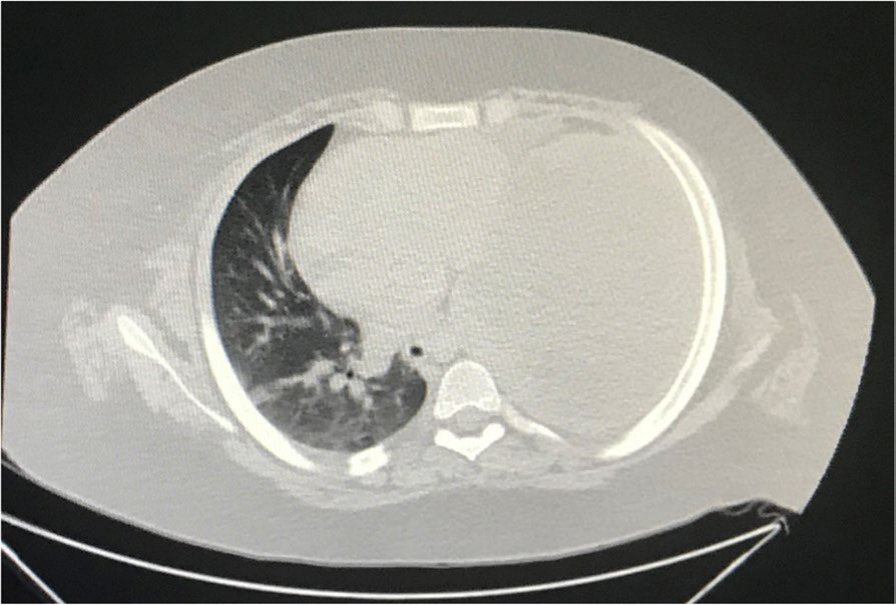
Emergency Chest High resolution computed tomography scan cut, showing severe empyema and complete collapse of the left lung in a 12-year-old patient with coronavirus disease

Due to the patient’s condition and suspicion of pulmonary collapse, an emergency computed tomography (CT) scan was done for the patient which showed severe left side empyema, plus the complete collapse of the left lung. (Fig. 1.) Therefore, the patient was scheduled for a pleural tap under the guide of sonography, but on account of his obesity, he was instead transferred to the operating room and a chest tube was inserted for him. The result of the patient’s pleura tap was the discharge of nearly 1.5 l of purulent liquid which was suggestive of empyema.

Results from the patients’ plural tap included a total cell count of 125,000 cells/ml, including 123,000 W.B.C/ml, along with 12 mg/dl sugar, 2.44 mg/dl protein, LDH level of 16,139 U/ml, and albumin of 3.3 mg/dl. The concurrent serum LDH levels was 644 U/ml, all of which points towards an exudative process.

**Fig. 2:**
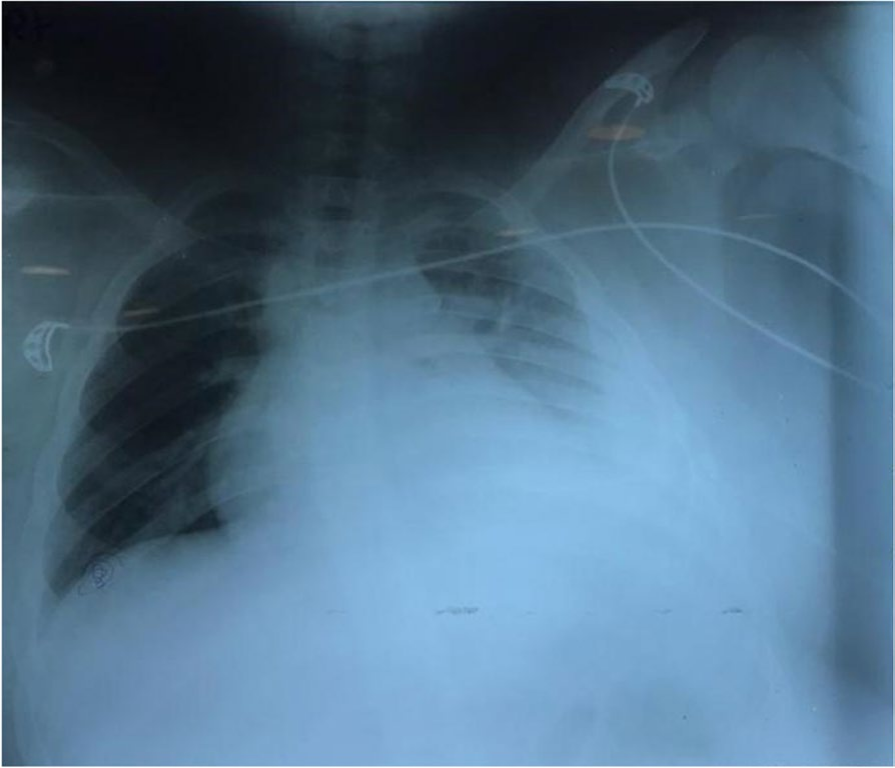
Chest X-ray after insertion of the Chest tube

A chest X-ray was done for the patient after his chest tube insertion to evaluate the position of the inserted chest tube and the remaining fluid. (Fig. 2.) After chest tube insertion, the patients’ respiratory distress resolved and non-invasive ventilation with an O_2_ mask was administered.

**Fig. 3:**
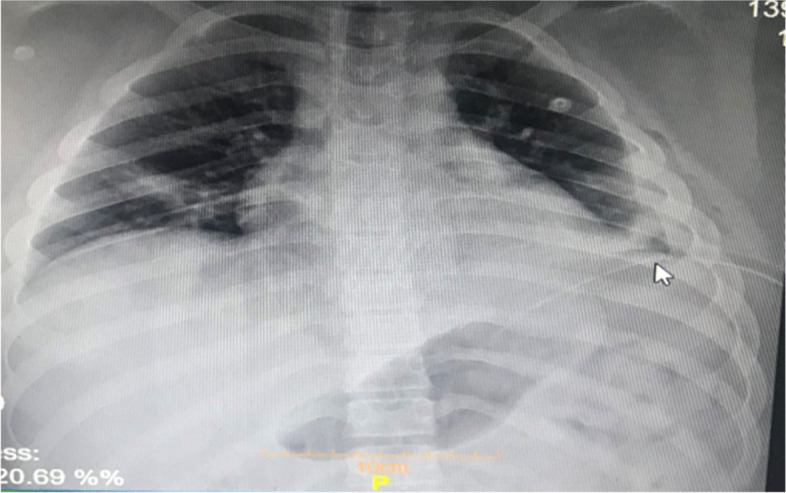
Chest X-ray after insertion of the Pneumocath (illustrated with pointer)

In his hospital course, intravenous vancomycin (1 g Q12h × 10 days) and meropenem (1 g every 8 h for 10 days) were started for the patient as empiric therapy for empyema. After four days, due to the absence of drainage from the chest tube and the improvement of the patient’s condition, the chest tube was removed. After 24 h from the removal of the chest tube, the patient once again started showing dyspnea and unstable vital signs. Based on the patient’s obesity, and the fact that he did not have proper respiration, this time a Pleural drainage needle catheter (Pneumocath) was inserted for him, followed by another chest X-ray for evaluation of the procedure. (Fig. 3).

Based on the patient history, clinical and paraclinical evaluation, a nasopharyngeal swab test for SARS-CoV-2 PCR was requested in which confirmed the diagnosis of COVID-19.

The patient’s notable laboratory data in his hospital course included a negative culture of the blood and pleural tap, a slightly increased Aspartate transaminase (AST) (45 IU/L), even higher amounts of W.B.C (25.51 × 10^3^/mm3) with a lymphocyte differentiation of 7% (lymphopenia), and Plt (685 × 10^3^/mm3) counts, and a normal level of ESR (5.5 mm/h). D-dimer also increased during his hospitalization, from an initial normal result of 0.3 μg/ml to 0.6 μg/ml. Other test results including Troponin levels, Blood coagulation tests, and other liver function tests were normal.

After 10 days of hospitalization, following antibiotic therapy and supportive care, the patient was discharged with oral antibiotics (Co-Amoxiclav 625 every 8 h for 7 days). His condition on discharge was stable with complete recovery during his follow-up.

## Discussion and conclusion

Pleural effusion is one of the rare complications of the COVID-19. According to previous research, it has been determined that pleural effusion does not occur at the onset of the symptomatology, and the risk of developing pleural effusion increases with the progression of the disease [23]. Empyema is defined as the presence of pus in the pleura space. The main cell covering the pleura space is the pleura mesothelial cell, which is activated in the presence of organisms and subsequently initiates the inflammatory response by releasing cytokines [24]. The pleura responds to the presence of microorganisms via a change in the permeability of the pleura that leads to exudative pleural effusion which is associated with the oozing of protein and white blood cells such as neutrophils [25]. The most common etiology of empyema is tough to be the infection of parapneumonic effusion infection which happens in almost half of the cases. This process usually resolves with antibiotic therapy and drainage, with limited side effects. However, diagnosis and following the progression of empyema is critical and affects the outcome of treatment, given the fact that no guideline for the management of empyema in the context of COVID-19 still exists [26].

As mentioned in our case, the patient had a three-week history of upper respiratory infection and COVID-19 related symptoms, which subsequently developed a superimposed bacterial infection. Also, our patient was very obese and had a high BMI based on his age, which caused inadequate ventilation and restricted the expansion of the lungs, which in terms attributed to the collapse of the lung, and development of infection and empyema. Furthermore, studies have shown that patients with obesity and morbid obesity are disproportionately affected with a severe form of COVID-19 [27, 28]. Obesity can limit ventilation by impeding diaphragm excursion, while also impairing immune responses to viral infection, [29] and induces oxidant stress to adversely affect cardiovascular function [30]. Kass et al. [28] reported that COVID-19 will affect younger populations more than previously reported in populations with a high incidence of obesity. The prevalence of severe COVID-19 disease should be reduced by public messaging to younger adults, maintaining greater vigilance for this at-risk population, and lowering the threshold for virus testing in obese individuals. The possibility of direct infection of adipocytes by the SARS-CoV-2 and a subsequent exaggerated inflammatory response could explain the pathogenic role of obesity in the severity of COVID-19 infection [31, 32].

The incidence of pleural empyema among children has increased over the last two decades [33]. Factors that cause complicated pneumonia or empyema are still uncertain. Previous research has shown that children who developed empyema were older, had longer febrile diseases, and probably have received ibuprofen or antibiotics before hospitalization [34]. Studies have indicated that recent varicella and pneumococcal infection are in association with the risk of pleural empyema formation [35]. In the aspect of developing pleural empyema in the context of viral respiratory illness, Crow Amoco et al. observed an increase in the number of children hospitalized for pleural empyema during the H1N1 outbreak in the spring and summer of 2009. Moreover, *Streptococcus pneumonia* and *Streptococcus pyogenes* were the agents that cause secondary bacterial infection [36]. Several other studies have reported the rise in cases of pleural empyema in a patient suffering from COVID-19 too, although the majority of them occurred in adults [23, 26].

In a study performed at New York University Langone Health in patients suffering from COVID-19, about 0.7% of patients had thoracic complications requiring surgery in which one of them (8%) developed with empyema which decortication was done and subsequent operative cultures demonstrated growth of Klebsiella pneumonia [37]. Moreover, Tessitore et al. described three cases of thoracic empyema followed by COVID-19 infection in the adult population due to superimposed infections with *Finegoldia Magna* species and *Pseudomonas aeruginosa* [26]..

In conclusion, pleural empyema should be considered as a rare complication of COVID-19. Since there is still no guideline in the management of empyema in the context of COVID-19, delay in diagnosis and intervention may cause morbidity and mortality in children. Pneumonia, either caused by COVID-19 or not, commonly manifests with pleural effusion; However, pleural effusion can be superimposed with a bacterial infection and result in empyema, which is a life-threatening entity if not diagnosed or managed properly, and based on its severity, requires antimicrobial therapy and surgical interventions. The golden key is prompt diagnosis and rapid management of these patients. In our case, the pleura drainage culture was negative, which could be due to the previous use of antibiotics. In COVID-19, the patients’ signs and symptoms should be properly addressed and managed, however, an important feature in managing these patients is the proper follow-up and observation of COVID-19 patients even after their acute phase of the disease, due to more latent complications (e.g. renal failure, diabetes, vasculitis, etc.) and in cases where complications such as respiratory distress, nasal flaring, dyspnea, or O_2_ saturation drop occur, proper evaluation and examination should be carried out to avoid any further complication and provide the most efficient management.

## Data Availability

All data regarding this study has been reported in the manuscript. Please contact the corresponding author if you are interested in any further information.

## Abbreviations

AST: Aspartate transaminase
BMI: Body Mass Index
COVID-19: coronavirus disease
CRP: C-Reactive Protein
CT: Computed tomography
PLT: platelet
ESR: Erythrocyte Sedimentation Rate
SARS-CoV-2: severe acute respiratory syndrome coronavirus-2
W.B.C: White Blood Cells
VBG: Ventricular Blood Gas
